# Dr. Marshall Hall’s Observations on the Prevention and Treatment of Apoplexy and Hemiplegia

**Published:** 1842-06-25

**Authors:** 


### ANALECTA.

[Apoplexy is a thread bare subject, but still often misunderstood ; we agree with Dr. Hall that the causes of it require to be looked into, in order to derive any good rules of treatment, and especially of prophylaxis. But in examining the causes of apoplexy, we must always bear in mind that several very different pathological conditions are attended with sudden loss of consciousness. If we restrict the term apoplexy to cases of vascular congestion or actual hæmorrhage, there is always positive disease in the vessels of the brain. This may arise from disease of the heart, from general fulness of the vascular system, or plethora, or from diseases of the vessels of the brain, or even of its substance.

In other cases the loss of consciousness is connected with diminished action in the vessels of the brain ; and those cases often arise in anemic or cachectic conditions of the system, or follow dyspepsia and liver disease. The treatment of these forms requires no little modification, and the rule which requires free blood-letting in apoplexy can only be admitted with numerous modifications. W. W. G.]

At a meeting of the Medical Society of London, April 4, 1842, Dr. Marshall Hall read a paper, entitled,

### *Observations on the Prevention and Treatment of Apoplexy and Hemiplegia.*

He commenced by saying, that in bringing the important subject of this paper before them, he had no hope that he could offer anything new. His object was rather to seek the information which its free discussion by the members of this society could not fail to elicit.

The question of the causes, nature, prevention, and treatment of apoplexy and hemiplegia was a very complicated one. He thought the attention of physicians, in reference to the prevention and treatment of apoplectic and hemiplegic attacks, had been far too much confined to the question of plethora as the disease, and of depletion as the remedy. It was to him certain that such attacks might and did occur quite irrespective of general plethora ; nay, that they occurred in connection with the opposite condition of the system, that of inanition and anæmia. Nor was a state of anæmia the only other condition besides plethora which led to the apoplectic or hemiplegic attack. Morbid conditions of the stomach and morbid conditions of the intestines were other sources of these seizures. But he had also observed the occurrence of apoplectic affections under other circumstances: other indubitably predisposing causes of the apoplectic seizure were dyspepsia, cachexia, and gout. Nor was even this view of the subject sufficiently extended ; the liver and the kidney must do their office. These sources of the apoplectic or hemiplegic seizure consisted in conditions of the general circulatory system, and of the blood itself. There were still others of a different kind.

The first of these was disease of the heart; and this consisted, first, in hypertrophy, with augmented impulse given to the arterial blood; or, second, in dilatation of the heart and disease of its valves, impeding the reflux of the blood along the veins.

The second was disease of the capillary vessels, of the minute arteries, or of the minute veins of the brain and its membranes.

Lastly, there were causes of apoplexy in the muscular efforts, by which the action of the heart itself was augmented, as in violent running, the ascent of a mountain, &c., and in other muscular efforts, by which the return of venous blood was impeded, as the efforts of vomiting, or for the expulsion of the fæces; and still more, of parturition.

This view of the causes of apoplexy would sufficiently denote the complexity of the problem of the prevention and treatment of the apoplectic and hemiplegic attack; for that prevention depended on restoring the system to a state of what may be termed equilibrium, in regard to plethora and inanition; to the removal of irritating or morbid matters from the primæ viæ; to the correction of the morbid diathesis in dyspepsia, gout, and cachexia. The prescription must include remedies and regimen to meet all these circumstances, and, as he had stated, the problem was by no means either an easy or a simple one. Yet another element in the problem was that which related to the local or topical remedies. On each of these sources of the apoplectic and hemiplegic attack, he proposed to make a few observations. These observations would be principally addressed to the medical practitioner; but as far as they might relate to regimen, they might, he thought, be profitably considered by the patient.

I. *Of Plethora, or Fulness.*—This cause of the apoplectic or hemiplegic seizure was that which had received most attention, or rather it was that *towards* which medical opinion was most biassed, not to say prejudiced. It was unnecessary for him to describe the symptoms of this condition so well known. The most satisfactory mode of treatment was to open a vein and allow the blood to flow from an ample orifice, the patient being placed in the perfectly erect position, until incipient syncope was induced: the quantity of blood which thus flowed was the diagnosis and measure of the disease in every respect. If the patient were young and robust, if the plethora were decided, and especially if there were real congestion and no laceration of the brain, a large and proportionate quantity of blood would flow before the slightest degree of syncope was manifested. No other measure afforded at once such security to the patient, and such information to the physician. It was impossible for him to speak in too high terms of the advantage of this measure in both these respects. In reference to blood-letting, there was this important question, Was the case one of *congestion* or *pressure*, or was there actual hæmorrhage with *laceration* of the substance of the brain? In the former case much blood would flow before incipient syncope occurred, and much might be, must be taken; but in the latter the injury had inflicted a shock upon the system, and little blood flowed before syncope appeared; and even the loss of that little was difficultly borne. To take more would be death! It might be said that we ought to distinguish the two cases *à priori*. He replied in the words of Celsus, "Id votum est." Turgidity and flushing denoted congestion, and pallor and collapse might denote laceration. But many cases occurred in which nothing so marked was observed; and in these, in the absence of an earlier and more perfect diagnosis, he knew by experience that the plan of instituting blood-letting proposed, afforded most important and salutary information, leading us on to take more blood in the case, in which greater depletion was required, and checking our depletion in those in which it would not be either well borne or remedial. But having made and repeated this statement on other occasions, and the profession being, he believed, well acquainted with it, he proceeded at once to another topic.

II. *Of Inanition.*—It was constantly his lot to see patients who were in jeopardy not from fulness but from inanition, and who had long been kept in a state of anæmia by blood-letting, general or topical, when an opposite treatment was required to restore the equilibrium of the system, and to remove the vertigo and other symptoms threatening an attack of apoplexy. A state of pallor, a disposition to faintishness, palpitation and nervous timidity, the occurrence of the symptoms when the stomach was empty, when the bowels had been relieved, and on suddenly looking upwards, or resuming the upright position on rising from bed, or after stooping, or the recumbent position: such were the diagnostic *signs* of a state of inanition from a state of plethora. The *history* of the case also afforded a diagnosis; for, although depletion might have appeared to afford a momentary *relief* of the symptoms, it had issued in their *aggravation* in general. An opposite mode of treatment, very cautiously and prudently adopted and pursued, would confirm the diagnosis, by affording a more permanent, though possibly a less immediate and marked relief. It was to the important distinction between the immediate and permanent relief, indeed, that he would draw the attention of the profession. In the case of symptoms portending apoplexy or hemiplegia, although these might arise from inanition, yet they were invariably *relieved* by depletion, although they afterwards returned with augmented force. This effect was very puzzling to the inexperienced practitioner. It was explained by the fact, that the symptoms ceased under the influence of a condition allied to syncope, but returned with the reaction. This subject must be carefully studied, in order that the nature and treatment of the case might be understood. He had next particularly to notice that the state of anæmia was not one of safety. In such circumstances apoplexy and hemiplegia, with the actual effusion of blood into the cerebrum, had occurred. Such a case was related by the late Dr. Denman: it occurred in the midst of exhaustion and anæmia from protracted uterine hæmorrhage; a clot of blood was found in the cerebrum. A similar case was detailed by Mr. Travers. This latter occurred under the actual use of the lancet, and during the flow of blood from the arm. A third case occurred to Mr. Hammond, of Brixton, after parturition: the patient was attacked with hemiplegia; she gradually recovered. We might, therefore, incautiously bleed our patients into apoplexy and hemiplegia! This statement should lead us to be very wary in the use of this remedy in doubtful or protracted cases. Even in cases of injury of the brain, as in concussion, the same question presented itself. This point was admirably illustrated by the following remark of Sir Benjamin Brodie:—"Where bleeding has been carried to a great extent, symptoms frequently occur which in reality arise from the loss of blood, but which a superficial observer will be led to attribute to the injury itself, and concerning which, indeed, it is sometimes difficult even for the most experienced surgeon to pronounce, in the first instance, to which of these two causes they are to be referred. Repeated copious blood-letting is of itself adequate to produce a hardness of the pulse, which we shall in vain endeavour to subdue by persevering in the same system of treatment. In many individuals it will produce headach and confusion of mind, not very different from what the injury itself had previously occasioned." The pallor of the countenance, the effects of position, the effects of fasting or of an active purgative, the history of the case, must be carefully considered in forming our diagnosis. The treatment would then consist in carefully restoring the system to its state of equilibrium.

III. *Of Dyspepsia and Cachexia.*—There could be little doubt that in dyspepsia the blood itself became contaminated, and, as it were, *cachectic;* on this principle we accounted for the appearance of furunculus and paronychia; for the morbid condition of the tongue and interior of the mouth, the general cutaneous surface, the secretions, &c. He had so often observed symptoms threatening the apoplectic or hemiplegic attack, in conjunction with symptoms of dyspepsia and cachexia, that he had no doubt of the vast importance of a strict attention to this subject. That very day (Oct. 1, 1841,) he had been consulted by a medical gentleman from Birmingham under these circumstances. One form of this affection was the following: vertigo occurred with faintishness, sickishness, and a cold clammy perspiration; sometimes there was actual sickness, sometimes much flatus. In these cases the feet and other extreme parts were apt to be cold. The secretion of the liver was frequently defective, and the urine was apt to deposit the lithic acid salts. Nothing could be so injurious as blood-letting. In no case was the loss of blood repaired with such difficulty. The application of a few leeches frequently left a state of debility and pallor which were felt and seen for weeks. The treatment consisted in the correction of the secretions, and in the infusion of tone and general health into the system. The compound decoction of aloes, the infusion of rhubarb, of gentian, of cinchona, singly, or better, mixed together; sarsaparilla; the vinum ferri; the bicarbonate of potass, stomachics, tonics, and antacids, in a word, were the principal internal remedies. But with these a mild, nutritious diet, a system of gentle exercises, early hours, the tepid salt-water shower-bath, and a strict attention to the condition of the feet and general surface, by means of the flesh-brush, flannel, and a frequent change of shoes and stockings, should be conjoined. Those engaged in the harassing affairs of a London life should sleep in the country, and cherish the utmost quiet of mind.

IV. *Of Gout.*—But he had frequently traced a connection between gout and its frequent attendant, the lithic acid diathesis, and the apoplectic and hemiplegic seizure. It was not merely plethora, or the opposite state of inanition, which led to the apoplectic attack. The morbid state of the blood in dyspepsia and cachexia also disposes, as he had already said, to this affection. The same remark applied to the condition of the system and of the blood, especially in gout; and, as he should have to observe immediately, the same disposition obtained in several morbid conditions of the liver and kidney. A nobleman, now no more, suffered in succession from gout and the herpes zoster, and the urine deposited the lithites copiously. He was relieved by the appropriate remedies, and became affected with an apoplectic (or epileptic) attack. A similar attack (without hemiplegia) occurred several months afterwards, and a third attack proved fatal. This gentleman was pallid, the prolabium being white. A steady perseverance in such remedies as the decoctium aloes compositum, the bicarbonate of potass, and the vinum ferri, had in other cases effectually averted the threatened evil. But he must make another remark. The vinum colchici should be given in very minute doses, as five drops thrice a-day, also steadily and persevering to overcome the specific gouty diathesis. The lithic acid diathesis was not the only urinary disorder which led to apoplexy and hemiplegia. This attack, it is well known, occurs in the case of diabetes and in that of albuminous urine. Although he had designated the attack apoplectic and hemiplegic, it was sometimes more allied to epilepsy than apoplexy. The gentleman to whose case he had briefly adverted, was affected with minute ecchymosed spots on the forehead, which he had only observed under three circumstances, viz., after severe vomiting, the effects of parturition, and the epileptic attack; when he saw him soon after the second seizure, the insensibility had passed away, and there was no hemiplegia.

V. *Of Disease of the Heart.*—It had long been supposed that disease of the heart is a cause of the apoplectic seizure, and hypertrophy of that organ had been fixed upon as the most influential in this respect. On this question the pathologists of France were much divided. Of the two latest writers on the subject, M. Andral was of opinion that hypertrophy was really a frequent cause of apoplexy; whilst M. Louis was of the opposite opinion. There could be no doubt that, *cæteris paribus*, hypertrophy of the heart would cooperate in inducing the apoplectic attack; but he thought that a much more energetic cause of apoplexy, and of congestion and hæmorrhage in general, was that form of disease which impeded the return of the venous blood from the brain, viz., dilatation and valvular disease. The worst form of hypertrophy might be unattended by symptoms or appearances of congestion; but no severe case of dilatation or of valvular disease ever existed, without lividity of the countenance, dozing, and other appearances and symptoms of apoplectic tendency. Altogether, however, we wanted a series of cases carefully taken and analysed, and statistically given, to *establish* the truth of the real influence of disease of the heart in inducing the really apoplectic seizure.

VI. *Disease of the Capillary and Minute Vessels.*—The influence of this cause of apoplexy was placed beyond question by post-mortem examination. Sometimes the morbid appearance was a dilated condition of the capillaries; sometimes an ossified condition of the minute arteries (?); sometimes a minute aneurism. Another important topic was that of "ramollisement," or softening of the brain, as the *cause*, and as the *effect* of the apoplectic or hemiplegic seizure. In resuming the subject he might remark, that it was not plethora alone which predisposed to the apoplectic and hemiplegic attack; the very opposite condition of the system, or anæmia, whether it arose from the loss of blood by blood-letting, or hæmorrhage, or from defective sanguification, was not free from this danger; dyspepsia and cachexia, as they induced external disease, as seen in furunculus, paronychia, &c., might also induce a paralytic affection, a morbid condition of the blood taking the place of plethora or anæmia.

VII. *Of Muscular Efforts.*—He might make the same remark in regard to muscular efforts, which he had done in regard to disease of the heart—those efforts which opposed resistance to the reflux of the venous blood, were much more efficient causes of the apoplectic seizure than those efforts which augmented the momentum of the arterial blood. Thus we rarely heard of the occurrence of apoplexy during the violence of the race, during the ascent of mountains, &c., but such an occurrence at the water-closet was by no means uncommon; and we all knew how apt the parturient efforts were to induce congestion of the brain, and the consequent apoplectic seizure. It would be most interesting to correct our ideas on these subjects by a cautious appeal to facts.—*London Lancet.* April 23, 1842.

